# Color‐Tunable Glass for Adaptable Thermal Management Based on Silver Phase Change

**DOI:** 10.1002/advs.202505791

**Published:** 2025-06-10

**Authors:** Mi Jin Hong, Seon Kyeong Kim, Jung Mi Im, Ik Hoon Jeong, Min Ju Kim, Jeong Jin Kim, Yeong Jae Kim, Gil Ju Lee

**Affiliations:** ^1^ School of Electrical and Electronics Engineering Pusan National University 2, Busandaehak‐ro 63 beon‐gil, Geumjeong‐gu Busan 46241 Republic of Korea; ^2^ Ceramic Total Solution Center Korea Institute of Ceramic Engineering and Technology 3321, Gyeongchung‐daero, Sindun‐myeon Icheon‐si Gyeonggi‐do 17303 Republic of Korea

**Keywords:** Ag nanoparticles, anti‐fogging, photothermal heater, plasmonic resonance, radiative cooler

## Abstract

In recent years, passive thermal management systems have emerged as an essential energy‐saving strategy to mitigate carbon emissions. However, traditional heat management approaches, such as photothermal devices and radiative coolers, often face application limitations due to their complex construction, aesthetic constraints, and inability to adapt to diverse architectural requirements. This study proposes a colored thermal engineering glass fabricated through a simple annealing process, where Ag nanoparticles are formed on a TiO_2_ film and an Ag layer. The structure of Ag layers can be easily controlled by adjusting the annealing temperature, allowing for systematic tuning of the optical efficiencies in the visible and long‐wave infrared regions. Consequently, thermal management devices can selectively function as either a photothermal heater or a radiative cooler, depending on the morphology of Ag layers. In particular, the photothermal device can function as an anti‐fogging solution for structural designs without requiring external energy. Furthermore, the stacked metal‐insulator‐metal structure induces plasmonic resonance phenomena, producing visible colors that range from vivid to dull hues. The tunable color variations enhance the suitability of the thermal management device for a wide range of architectural applications.

## Graphical abstract

This article presents an Ag particle‐based thermal management device achieving both selective visible reflection and effective heat flux control. Morphological changes of silver during annealing enable tunable color and thermal regulation via a simple fabrication process. Depending on the Ag state, the device provides radiative cooling in the open state and photothermal anti‐fogging functionality in the closed state.

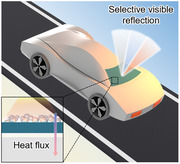

## 1 Introduction

In recent years, the climate crisis has emerged as a critical global issue, primarily driven by the continuous rise in greenhouse gas emissions.^[^ ^1^, ^2^, ^3^, ^4^, ^5^ ^]^ A major contributor to carbon emissions is the excessive energy consumption required for heat regulation, such as heating and air conditioning systems.^[^ ^6^, ^7^, ^8^ ^]^ In particular, vehicles and buildings consume excessive energy to maintain comfortable indoor environments through heat regulation.^[^ ^9^, ^10^ ^]^ The significant increase in atmospheric greenhouse gas concentrations has highlighted the importance of developing eco‐friendly and sustainable sunlight‐harvesting technologies to address environmental challenges.^[^ ^11^, ^12^, ^13^ ^]^ Ongoing efforts to develop renewable energy solutions have focused on reducing energy consumption through various solar‐driven strategies, including photothermal devices and radiative coolers.^[^ ^14^, ^15^, ^16^, ^17^ ^]^ The long‐wave infrared (LWIR) spectrum is broadly utilized for heat flux management as this region corresponds to the thermal radiation emitted by most objects at typical environmental temperatures. The Earth's atmosphere features a transparent electromagnetic window spanning 8–13 µm in the LWIR region, which enables efficient thermal radiation to escape into outer space.^[^ ^18^, ^19^ ^]^ Therefore, thermal management devices utilizing this region effectively regulate heat flux without requiring additional energy sources. Notably, photothermal heaters enhance the surface temperature while preventing heat dissipation into outer space through LWIR reflection.^[^ ^20^, ^21^, ^22^ ^]^ The near‐zero emissivity in the LWIR region helps maintain indoor temperatures in architectural structures under various environmental conditions. On the other hand, radiative coolers function as passive cooling systems that dissipate heat by emitting infrared radiation through the atmospheric window, allowing objects to cool without input energy.^[^ ^23^, ^24^, ^25^ ^]^ However, most research on passive thermal regulation systems has been limited in application due to sophisticated construction and aesthetic constraints.^[^ ^26^, ^27^, ^28^, ^29^, ^30^ ^]^

Therefore, we propose a simple and effective heat management system based on Ag particle‐based thermal management (APTM) systems, in which a TiO_2_ layer is positioned between thin Ag layers. The fabrication process of APTMs consists of a straightforward e‐beam evaporation and annealing procedure, which can be scaled up for large‐area production due to the simplicity of the process. During annealing, Ag films within the APTM are transformed into nanoparticles (NPs) through a spheroidization mechanism, which is strongly affected by the annealing temperature and duration.^[^ ^31^, ^32^, ^33^, ^34^ ^]^ The samples were annealed at different temperatures: 400, 600, and 1000 °C. Since both Ag layers are influenced by the annealing process, the optical properties in the solar spectrum and LWIR region can be actively tuned.^[^ ^35^ ^]^ By modifying the optical characteristics in both spectra, our system enables both photo‐induced heating and radiative cooling. In particular, the dense Ag components in APTMs exhibit high reflectance in the LWIR region, reducing heat transmission from the outdoor environment to indoor spaces and minimizing heat loss from the interior. Conversely, APTMs with sparse Ag NPs produced at high temperatures, manifest the highest absorptance in the LWIR region. The high emissivity of sparsely distributed Ag NPs enables radiative cooling, primarily driven by the dominant effect of the glass substrate. Therefore, APTMs can be classified as Ag particle‐based photothermal heaters (APPH) or radiative coolers (APRC), depending on the distribution of Ag particles. Furthermore, the visible versatility of APTMs can be achieved by layering metal, insulator, and metal layers, which generate cavity plasmon resonance.^[^ ^36^, ^37^, ^38^ ^]^ Additionally, Ag NPs act as absorbers in a specific visible wavelength range due to surface plasmon resonance.^[^ ^39^, ^40^, ^41^, ^42^, ^43^ ^]^ The distinct structural characteristics of Ag layers make APTMs possess a range of colors from magenta to bluish colors. Therefore, APTMs offer advantages in cost‐effectiveness, scalability, as well as multi‐functional and aesthetic applications.

To verify optical efficiencies across broad wavelengths (i.e., from the solar spectrum to the LWIR region), we conduct 3D rigorous‐coupled wave‐analysis (RCWA) simulations. In addition, the Maxwell‐Garnett effective medium theory (EMT) is employed in optical simulations to determine the volume ratio of air voids within the bottom Ag layer. Component analyses such as Transmission Electron Microscope (TEM), Energy‐Dispersive X‐ray Spectroscopy (EDS), and X‐ray Diffraction (XRD) support the characterization of the structural and elemental properties of the APTMs. Scanning Electron Microscopy (SEM) further reveals the fill factor of Ag NPs for numerical analyses. Additionally, Multiphysics simulations and outdoor measurements confirm the optimum conditions for APPH and APRC, demonstrating remarkable thermal management capabilities. Specifically, the anti‐fogging experiments under humid conditions validate the outstanding heating performance of the APPH. A comparison of glass surfaces with and without the photothermal coating reveals a significant reduction in condensation and demonstrates the effectiveness of APPH in preventing fog formation. In conclusion, our photoactive thermal management systems revolutionize solar‐to‐energy solutions through their structural simplicity and optical flexibility.

## 2 Results and Discussion

### 2.1 Ag Particle‐Based Thermal Management Devices

**Figure 1:**
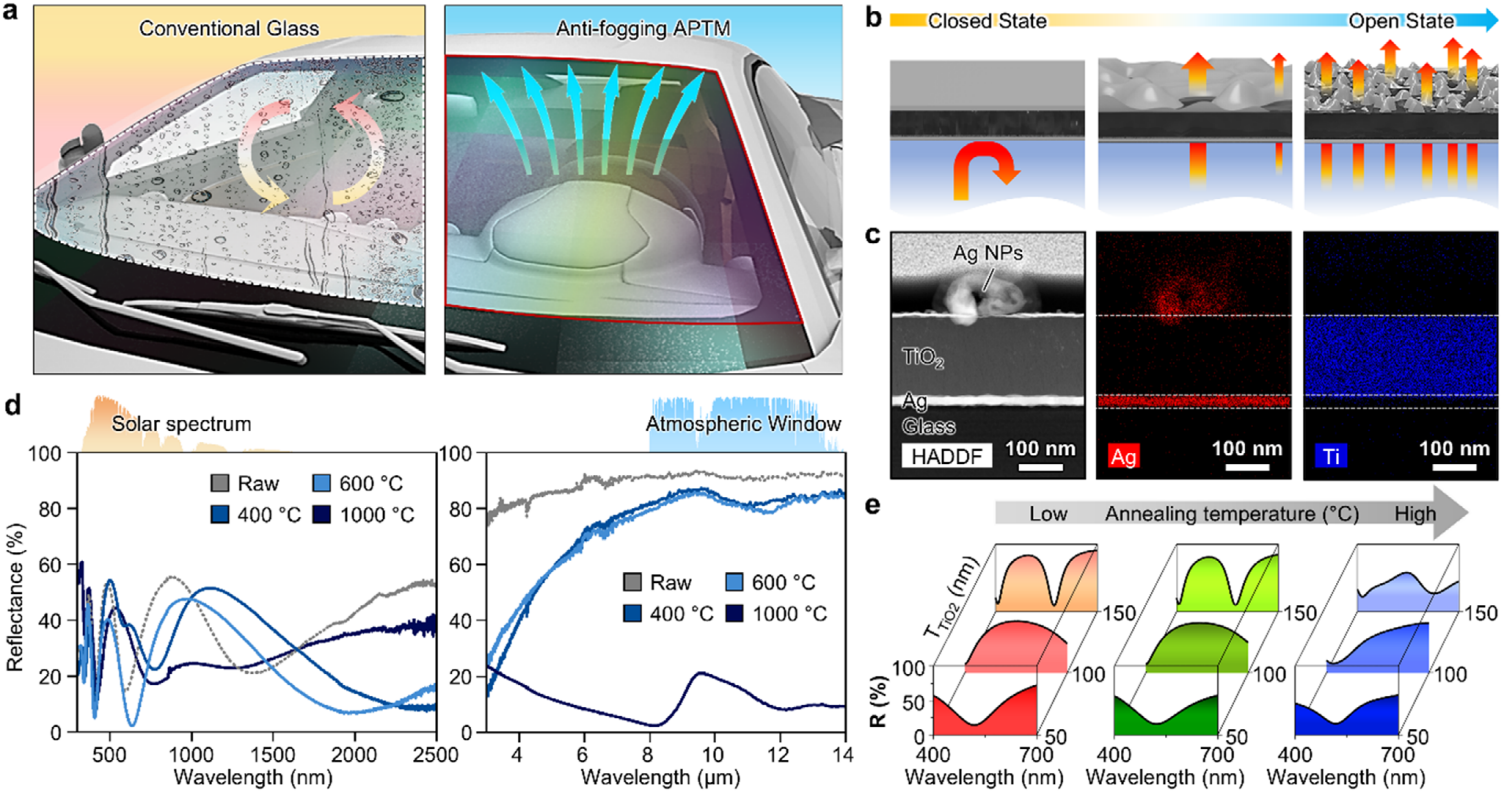
The Ag particle‐based thermal management devices. a) Schematic of the APTM laminated onto the front glass of a car for thermal regulation. Unlike conventional glass, APTMs offer effective anti‐fogging performance and quickly remove moisture. b) Illustration of long‐wave infrared (LWIR) light transmission as the structure changes from the closed state to the open state, where the porous Ag layers allow LWIR light to pass through. The optical characteristics change with fabrication variables, allowing the APTM to function as either a photothermal heater or a radiative cooler, depending on the morphology of the Ag layers. c) Structural and compositional analyses of the APTM based on TEM‐EDS results. The Ag component indicates that the Ag film transforms into spherical nanoparticles during the Ag dewetting process. d) Measured optical efficiencies of APTMs with 10 nm Ag layers across wavelengths from 0.3 to 2.5 µm and 3 to 14 µm, shown alongside the solar intensity spectrum. e) Visible properties as a function of the TiO_2_ film thickness and annealing temperature.

**Figure** **1**a presents a schematic of an Ag particle‐based thermal management device integrated into the laminated glass of a car. Unlike conventional glass, APTMs exhibit superior anti‐fogging capabilities by effectively preventing moisture condensation and facilitating rapid removal of water droplets from the surface. Figure 1b illustrates the tunable optical properties of the APTM according to the Ag layer configuration (i.e., thin films, porous structures, and nanoparticles), which is controlled by the annealing temperature during the Ag dewetting process. When exposed to an incident LWIR light, the system generates heat flux that is either blocked or absorbed by the underlying structures, depending on the morphology of the two Ag layers. These structural changes enable selective reflection in the visible range and alter LWIR interactions. To observe the structural and compositional properties of the APTM, we performed elemental analysis using TEM and EDS (Figure 1c). The TEM‐EDS results confirm the spherical formation of Ag NPs based on the Ag component. The configuration of both Ag layers can be adjusted by controlling the annealing temperature (Figure S1, Supporting Information). As the annealing temperature increases, the top Ag layer transforms into nanoparticles, while the bottom Ag layer is partially transformed into a meshed structure. This difference arises because the bottom Ag layer is sandwiched between the TiO_2_ layer and the glass substrate, where dewetting is suppressed.^[^ ^44^ ^]^ The sandwiched configuration hinders dewetting because Ag atoms experience stronger confinement and reduced surface mobility due to adhesion with both adjacent layers. In contrast, the top Ag layer with a single contact interface and an air‐exposed surface undergoes more rapid dewetting and nanoparticle formation.

Figure 1d shows the measured optical efficiencies of APTMs with 10 nm Ag layers, displayed with the solar spectrum and sky transmission spectra. The raw state refers to the APTM without annealing, while other conditions are determined by the annealing temperature (i.e., 400, 600, and 1000 °C). The raw, 400, and 600 °C states exhibit high reflectance in the LWIR region due to the presence of dense Ag particles on both sides. The high reflectance in the LWIR indicates the retention of internal heat despite changes in the ambient environment. On the other hand, the 1000 °C state exhibits low reflectance in the 8–13 µm range, where Earth's atmosphere permits radiation to escape into space. The low reflectance in the LWIR region correlates with high emissivity, enabling the device to dissipate heat efficiently as a radiative cooler. Figure 1e displays the reflectance of APTMs in the visible range (i.e., 400 to 700 nm), depicted with fabrication conditions. The reflectance rarely varies in the case of the 50 nm TiO_2_ despite increasing the annealing temperature. Therefore, the 150 nm TiO_2_ is selected as the optimal case since the reflectance peaks are the most distinguishable among each condition.

### 2.2 Fabrication and Structural Analyses of the APTM

**Figure 2:**
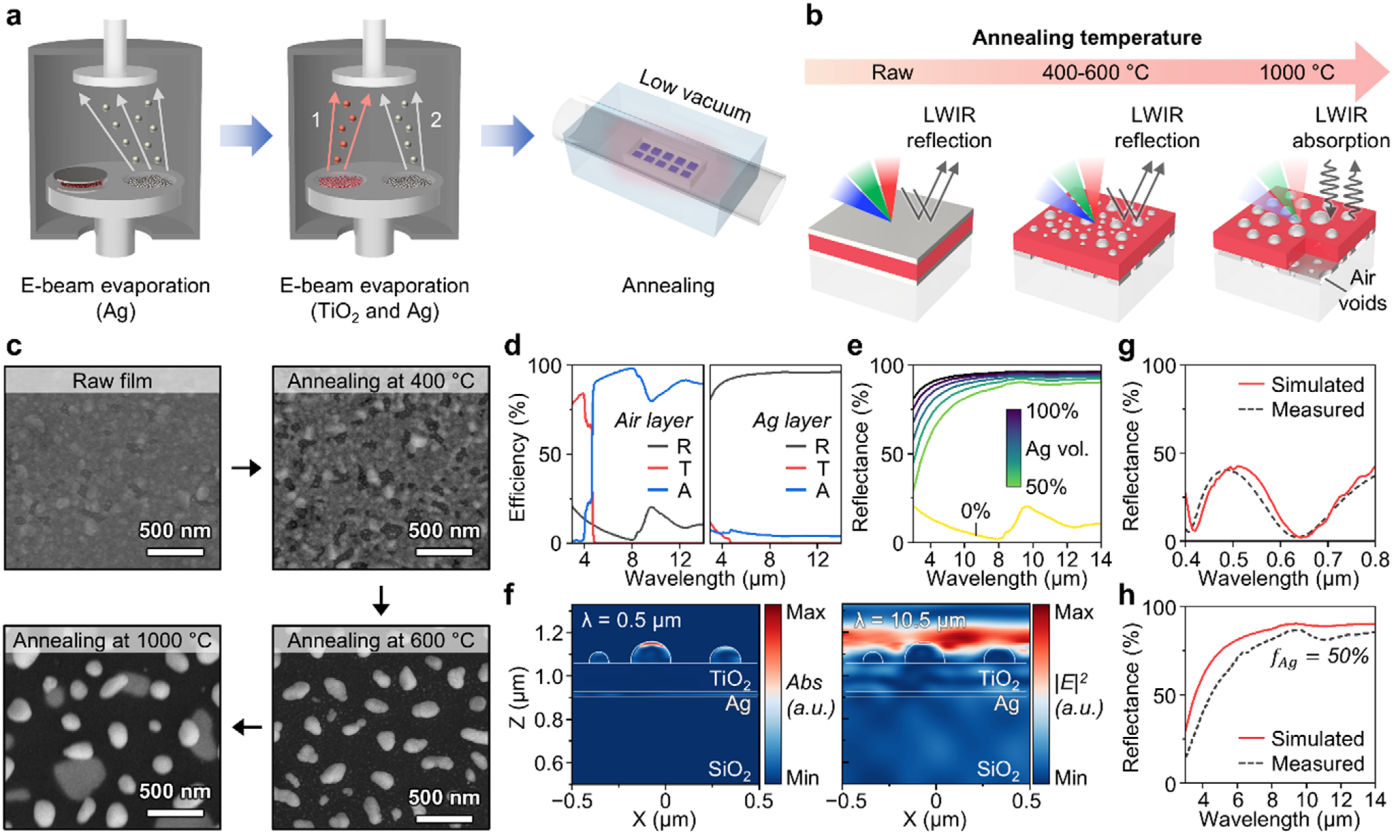
Straightforward fabrication process and optical efficiencies of APTMs. a) Schematic of the APTM fabrication process. The simple fabrication process of APTMs involves e‐beam evaporation and annealing. During annealing, the top Ag layer transforms into nanoparticles, while the bottom layer becomes a porous film. b) Illustration of APTMs as the annealing temperature increases. Initially, the TiO_2_ film is located between thin Ag layers. As the annealing temperature increases, the structure of the Ag layers changes, leading to muted or entirely shifted colors. Moreover, the reflective properties in the LWIR region, present in the raw, 400, and 600 °C states, shift toward absorption when annealed at 1000 °C. c) Top‐view SEM images of APTMs with 10 nm Ag layers. As the annealing temperature increases, the top Ag layer transforms into Ag nanoparticles. d) Simulation results when the bottom layer is composed entirely of air or a fully continuous Ag layer. e) Reflection variation according to the Ag volume fraction in the LWIR region. To account for the porous Ag film, the Maxwell‐Garnett effective medium theory (EMT) is applied. f) 2D contour maps of the electric and absorption fields in the visible and LWIR regions, respectively. g) Considering the Ag filling fraction, the optical efficiencies of an APPH (i.e., the APTM annealed at 600 °C with a 10 nm Ag layer) are depicted along with the measured results from a UV–vis‐NIR spectrophotometer h) and FT‐IR.

**Figure** **2**a illustrates the fabrication procedure of the APTM. First, a soda lime glass substrate is cleaned in an ultrasonication bath with acetone for 60 min, followed by rinsing with isopropyl alcohol (IPA) and N_2_ blowing. After cleaning the glass, the bottom Ag and TiO_2_ films are deposited by the e‐beam evaporator in sequence. Subsequently, the top Ag layer is repetitively evaporated through the same method. Both Ag layers have identical thicknesses of 3, 5, 10, and 20 nm. To control Ag formation on both sides, the annealing process is performed under low‐vacuum conditions. The annealing duration is fixed at one minute to optimize the temperature for Ag configuration. Owing to the simplicity and reproducibility of the fabrication process, large‐scale production is feasible over 2 inches (Figure S2, Supporting Information). Figure 2b displays the schematic and corresponding optical properties of the APTMs. At the raw state, the metal‐insulator‐metal stacked structure induces cavity plasmonic resonance and results in vivid visible colors.^[^ ^45^, ^46^ ^]^ Additionally, high reflection in the LWIR region is observed due to the dense Ag layers on both sides. The top Ag layer undergoes spheroidization as the annealing temperature increases, while the bottom Ag layer transforms into a meshed structure interspersed with air voids. The 400 and 600 °C states exhibit relatively muted colors and highly reflective properties in the LWIR spectrum due to the dense formation of Ag layers. In contrast, the 1000 °C state displays the dullest hues and high absorption of LWIR light instead of reflecting it, as the high temperature leads to the sparse Ag particles. Figure 2c represents top‐view SEM images of APTMs with 10 nm Ag layers, organized by the annealing temperature. While the raw and 400 °C states consist of densely packed Ag particles, the samples annealed at 600 and 1000 °C exhibit sparsely distributed Ag NPs. XRD results confirm that the annealing process does not affect the elemental properties of APTMs, as the chemical states of Ag and TiO_2_ are maintained up to 1000 °C (Figure S3, Supporting Information).

Accordingly, we conduct wave‐optics analyses to correlate the optical properties with structural parameters (i.e., fill factor and diameter) of Ag NPs. From the SEM images, the fill factor (FF) is derived from the ratio of the area covered by Ag NPs to the entire scanning region, which is obtained from the threshold images (Figure S4, Supporting Information). The FF values for the raw, 400, 600, and 1000 °C states with 10 nm Ag layers are 88.5%, 79.6%, 19.7%, and 14.9%, respectively (Figure S5, Supporting Information). At annealing temperatures above 400 °C, the FF sharply decreases by 60%, and Ag NPs are noticeably formed. The size distribution of Ag NPs in APTMs annealed at 600 and 1000 °C is analyzed, revealing that smaller and more uniform particles are formed at the lower temperature, with average diameters of 114 and 161 nm, respectively. The structural analyses of APTMs with 20 nm Ag layers are also shown in Figure S6 (Supporting Information). For 20 nm Ag layers, the raw, 400, 600, and 1000 °C samples exhibit FF values of 95.3%, 84.6%, 26.8%, and 15.0%, respectively. The thicker Ag layer tends to delay the onset of dewetting, resulting in a denser structure compared to the 10 nm case. Similar to the 10 nm Ag layers, the FF sharply decreases by more than 57.8% when annealed above 400 °C, and distinct Ag nanoparticles start to appear. However, irregular island‐like particles are observed instead of perfectly spherical morphologies at 600 °C. The number of particles analyzed in 10 nm‐thick Ag films is 222 and 137 for samples annealed at 600 and 1000 °C, respectively, while 20 nm‐thick Ag films exhibit 253 and 154 particles under the same conditions. Dewetting begins relatively later in the 20 nm samples due to the increased film thickness, resulting in a higher fill factor and larger Ag nanoparticles compared to the 10 nm cases.

Based on these results, a numerical study using RCWA simulation explains the light‐matter interaction at each wavelength (i.e., solar spectrum and LWIR range). The computational domain is designed to replicate the APTM devices, as depicted in Figure S7 (Supporting Information). The domain consists of Ag nanoparticles, a TiO_2_ layer, an EMT layer, and a soda lime glass substrate. To account for the effect of the bottom porous Ag layer on LWIR optical properties, the Maxwell‐Garnett EMT is adopted for the calculation. Ag is treated as a homogeneous host medium in the EMT layer, while air is considered a void.^[^ ^47^ ^]^ The detailed method is explained in Note S1 (Supporting Information). Figure 2d presents the optical efficiencies when the EMT layer is entirely composed of either air or Ag. The reflectance (R) is low when this layer consists of 100% air, whereas a fully Ag layer exhibits near‐unity reflectance in the LWIR region. To investigate the intermediate states, the air void filling fraction varies from 0% to 50%, corresponding to an Ag volume fraction ranging from 50% to 100% (Figure 2e). As the Ag fraction increases in 10% increments, the reflectance in the LWIR also increases. Moreover, 2D contour maps of the APTM with a 10 nm Ag layer annealed at 600 °C, one of the candidates for APPH, propose the absorption field and the electric field distribution in the *xz*‐plane at wavelengths of 0.5 and 10.5 µm, respectively (Figure 2f). At 0.5 µm, absorption selectively occurs at the surface of the Ag NPs due to size‐dependent properties corresponding to the surface plasmonic resonance (SPR) phenomenon.^[^ ^48^, ^49^ ^]^ However, the electric field penetration is mostly limited at a wavelength of 10.5 µm, suggesting minimized heat flux within the layers. Metal nanoparticles also contribute to increased absorption in the solar spectrum. A comparison of the absorption fields between the multifilm structure and Ag nanoparticles placed above the TiO_2_‐Ag layers at a wavelength of 2.0 µm shows enhanced solar absorption due to localized surface plasmon resonance (Figure S8, Supporting Information). Figure 2g,h display the spectral results of the APPH, plotted with the measured data from a UV–vis‐NIR spectrophotometer. In the visible region, the APPH has a reflectance peak ≈0.5 µm indicating a bluish color, whereas near‐high reflection is observed in the LWIR region. Based on the simulation results in Figure 2e, the APPH is assumed to have a bottom layer with an Ag porosity of 50%.

### 2.3 Color Variation of the APTM

**Figure 3:**
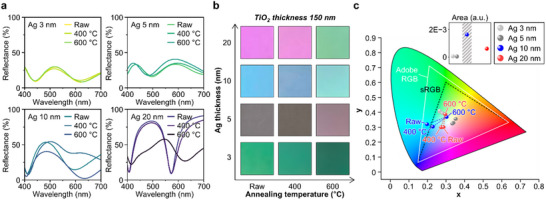
Aesthetic properties of APTMs. a) The reflectance results of APTMs measured using a UV–vis‐NIR spectrophotometer. APTMs with 10 and 20 nm Ag layers indicate distinctive optical characteristics in the visible region. b) Photographs of APTMs placed on a light‐blocking sheet. c) The CIE 1931 color space diagram with the color coordinates of APTMs. The graph above the color gamut displays the triangle area variation corresponding to changes in annealing temperature.

The APTMs exhibit color diversity in the visible region depending on the thickness of the Ag layers and the annealing temperature, as shown in **Figure** **3**a. When the Ag thickness is 3 or 5 nm, the reflectance at wavelengths from 400 to 700 nm does not change significantly. However, the reflection spectra show notable changes for Ag thicknesses of 10 and 20 nm, in which the reflection dip shifts with increasing annealing temperature, indicating color variation. Figure S9 (Supporting Information) presents the simulated reflectance results in the visible region as a function of TiO_2_ film and Ag layer thickness. The reflection peak position of the reflectance is determined by the TiO_2_ thickness, while the Ag thickness influences the bandwidth of the reflectance peak. Furthermore, the type of metal used in the cavity‐resonant system influences the vividness of hues (Figure S10, Supporting Information). Among various metal nanoparticles, Ag exhibits the most distinct reflective properties in the visible region, depending on the TiO_2_ thickness. Figure 3b displays photographs of APTMs fabricated under various conditions (i.e., Ag thickness on both sides and annealing temperature). The photographs were taken with a light‐blocking sheet placed behind the samples. Distinguishable colors are particularly observed for the 10 and 20 nm Ag layers, ranging from sky blue to yellow and magenta to green, respectively. As the annealing temperature increases, the vivid colors gradually fade or shift to entirely different colors. The color variation arises from cavity plasmonic resonance at specific visible wavelengths, induced by the metal‐insulator‐metal structure.^[^ ^50^, ^51^ ^]^ Moreover, Figure S11 (Supporting Information) indicates the photographs and spectral properties of APTMs annealed at 1000 °C in the visible region, indicating that over‐annealing leads to a broader spectrum peak. This is because excessively high annealing temperatures lead to the disappearance of Ag particles, which in turn reduces the vividness of the color.

To compare the color coordinates of each sample numerically, the color space system is introduced. The CIE 1931 color space represents colors visible to the human eye, which is used for mapping the color coordinates.^[^ ^52^, ^53^ ^]^ This system allows for a quantitative analysis of the color differences between various samples and provides a visual representation of point positions within the color gamut.^[^ ^54^, ^55^ ^]^ Figure 3c shows the color distribution for each condition in the CIE 1931 space, illustrated with the sRGB and Adobe RGB gamut, deciding the color reproducibility.^[^ ^56^ ^]^ The plot above the CIE 1931 diagram shows the triangle area of the coordinates concerning the annealing temperature (i.e., 400, 600, and 1000 °C). The value for APTMs with 10 nm Ag layers is 1.62E−3, while those for Ag thicknesses of 3, 5, and 20 nm are 4.78E−5, 4.32E−5, and 5.97E−4, respectively. From the results, we can conclude that APTMs with 10 nm Ag layers achieve the widest color saturation. Consequently, the color discrimination properties of APTMs can be tailored for diverse applications through color modulation in the fabrication process.

APTMs also exhibit angle robustness due to the scattering by randomly distributed nanoparticles and the presence of the TiO_2_ intermediate layer. Figure S12 (Supporting Information) shows the outstanding angle‐tolerant properties of the APTM based on simulation results. As the incident angle varies, the reflectance in the solar spectrum remains stable. Low reflectance above 1.5 µm indicates that absorption predominantly occurs within the nanoparticles. Furthermore, the presence of the intermediate TiO_2_ layer also enhances the isotropic colorimetric properties. A detailed explanation is provided in Note S2 (Supporting Information). To compare the angle tolerance of the samples, we fabricated APTMs designed to display a green color (Figure S13, Supporting Information). To match the green hue of the TiO_2_‐based sample, we deposited a 130 nm‐thick SiO_2_ layer between two 10 nm Ag layers. Compared to the sample composed of TiO_2_ layer, which maintains a relatively stable color regardless of the viewing angle, the sample with a SiO_2_ intermediate layer exhibits color variations depending on the angle. These findings highlight the angle‐independent color performance of TiO_2_‐based APTMs.

### 2.4 Long‐Wave Infrared (LWIR) Characteristics and Multiphysics Simulation

**Figure 4:**
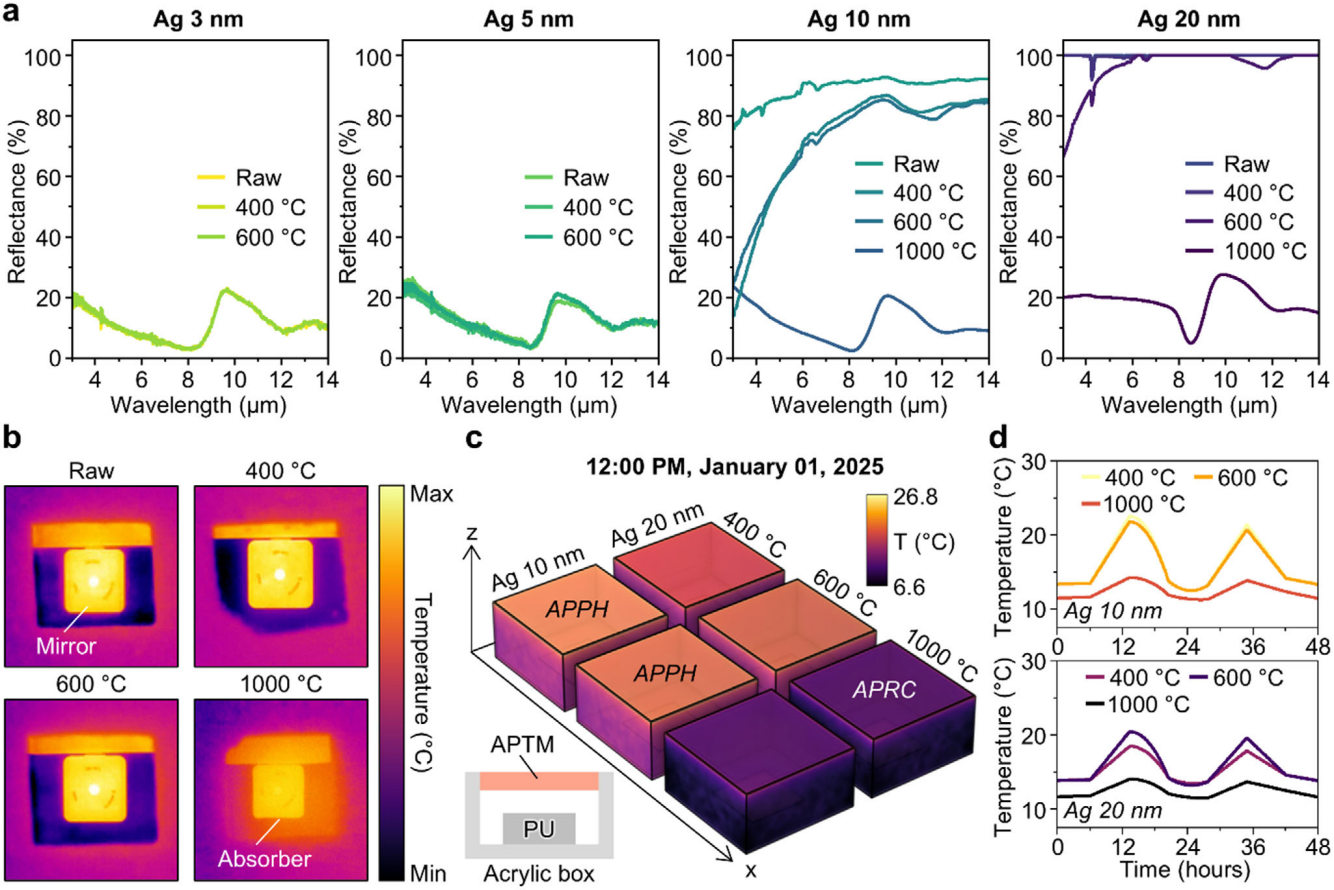
FT‐IR results and thermal simulation results of APTMs. a) FT‐IR results of APTMs as a function of Ag layer thickness. The reflectance does not vary significantly for ultrathin Ag layers (i.e., 3 and 5 nm), whereas the reflectance decreases noticeably as the annealing temperature increases for 10 and 20 nm Ag layers. b) Thermal camera images of APTMs with a 10 nm Ag layer. In contrast to the raw, 400, and 600 °C states, the sample annealed at 1000 °C shows the absorber property, consistent with the FT‐IR results. c) 3D temperature maps of APTMs obtained from thermal simulations. d) Temperature results from the upper side of the polyurethane (PU). APPHs exhibit the highest temperature relative to the ambient temperature, while the APRC shows the lowest.

The heat controllability can be predominantly determined by the optical characteristics in the LWIR region. **Figure** **4**a shows the Fourier Transform Infrared Spectroscopy (FT‐IR) results of APTMs according to the Ag layer thickness. APTMs with ultrathin Ag layers (i.e., 3 and 5 nm Ag layer) show no variation in reflectance with the increment of the annealing temperature, whereas APTMs with 10 and 20 nm Ag layers demonstrate significant differences in reflectance. In contrast to APTMs annealed at low temperatures, the 1000 °C states demonstrate low reflectance similar to the cases with ultrathin Ag layers. To further investigate the influence of asymmetric Ag thickness on optical characteristics, we additionally fabricated APTMs using combinations of 5, 10, and 20 nm Ag layers, as detailed in Note S3 (Supporting Information). Figure 4b confirms the optical properties of APTMs using a thermal camera. The raw, 400, and 600 °C states possess mirror‐like behavior, as the camera shape is clearly captured in the thermal images. Conversely, the sample annealed at 1000 °C shows reduced thermal intensity, indicating an absorbing property. Based on the measured data, we conducted a Multiphysics simulation using a heat transfer function to evaluate the thermal management performance of APTMs. The detailed simulation method is described in Note S4 (Supporting Information). Figure 4c presents 3D temperature maps under various APTM configurations. At 12:00 PM on January 1, 2025, in South Korea, when solar intensity reaches its peak during the day, the APTMs annealed at 400 and 600 °C with 10 nm Ag layers reach the highest temperature among various APTMs. Therefore, these conditions are most suitable for APPH operation. On the other hand, the APTM annealed at 1000 °C with 20 nm Ag layers exhibits the lowest surface temperature as both Ag layers contain sparse particles, which can function as the APRC. To evaluate the thermal response of the inner components, the temperature of the upper surface of the polyurethane (PU) is measured (Figure 4d). Over 48 h, APPHs exhibit the highest surface temperature, while the APRC shows the lowest.

### 2.5 Outdoor Measurements

**Figure 5:**
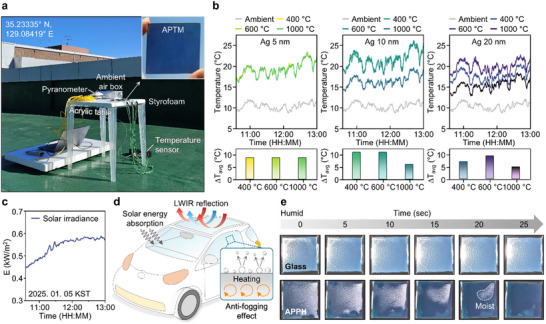
Outdoor measurements and anti‐fogging capability of the APPH. a) The outdoor measurement setup for evaluating thermal regulation performances. The configuration consists of an acrylic table, Styrofoam, samples, a temperature sensor, an ambient box, and a pyranometer. The temperature sensor is attached to the backside of the samples. The ambient temperature is also measured to compare the heating or cooling capabilities of the samples. b) Temperature results of APTMs: The samples annealed at 400 and 600 °C with 10 nm Ag layers exhibit the highest temperature difference, indicating their potential as APPHs. In contrast, the sample annealed at 1000 °C with 20 nm Ag layers shows the lowest temperature difference, functioning as the APRC. c) Solar irradiation intensity was measured from 10:30 AM to 1:00 PM on January 5, 2025, in Busan, South Korea. d) Schematic illustration of the APPH's potential application for anti‐fogging in vehicles, where the elevated temperature relative to the ambient environment helps remove humidity from the window. e) The defogging performance of the APPH is compared with bare soda lime glass.

Outdoor measurements are implemented to evaluate the thermal management performance of APTMs in real‐world conditions. **Figure** **5**a displays a photograph of the measurement setup, where temperature sensors are attached behind the samples. Styrofoam entirely covers the acrylic table to insulate against external heat. A pyranometer measures solar irradiance on a flat surface, providing data on the total shortwave radiation (i.e., 0.3 to 3.0 µm) from the sun and sky. To monitor the ambient temperature, an additional temperature sensor is positioned facing away from the sun to minimize external influences. The inset shows a magnified view of the APTM device. The temperature measurements taken between 10:30 and 13:00 on January 5, 2025, in Busan, South Korea, are plotted in Figure 5b. Figure 5c presents the solar irradiation intensity within the measurement duration. For 5 nm Ag layers in Figure 5b, the temperature variation exhibits minimal change with respect to the annealing temperature. This indicates that ultrathin Ag layers have minimal influence on the dewetting process, thereby resulting in static optical properties. However, APTMs with 10 and 20 nm Ag layers show significant variations in the temperature as the annealing temperature increases. Notably, APPHs (i.e., the sample annealed at 400 and 600 °C with 10 nm Ag layers) exhibit the highest temperature, manifesting an effective photothermal heating capability. The selection of optimal APPHs can be guided by the desired color of the sample, allowing for tailored applications. On the other hand, the APRC (i.e., the sample annealed at 1000 °C with 20 nm Ag layers) has the smallest temperature difference relative to the ambient temperature. Despite the presence of color, which prevents a temperature drop below the ambient level, the significantly lower temperature compared to other conditions enables efficient operation as the APRC. The differences in performance arise because optical performance in the solar spectrum can also influence heat management applications despite the similarity in LWIR properties (Figure S14, Supporting Information). To determine the absorbed solar power, the spectral absorptance ε_sample_(λ) of the sample is multiplied by the incident solar spectral irradiance I_AM1.5_(λ), based on the AM1.5 global standard. The resulting product is integrated over the solar spectrum to obtain the total absorbed power. The power density of solar irradiance for each condition is listed in Table S1 (Supporting Information). The orange‐colored APPHs exhibit power densities of 40.7% and 35.5%, while the sky blue‐colored APRC shows 14.2%. The APPH demonstrates high solar absorptance for indoor heating, whereas the APRC features low solar absorption for effective radiative cooling. Indeed, the observed color of the APTMs indicates partial absorption in the visible region, which diminishes the cooling performance, as shown in Figure S15 (Supporting Information). A trade‐off exists between aesthetic appearance and radiative cooling functionality.

In addition, the average reflectance in the LWIR region is summarized in Table S2 (Supporting Information). R_8‐13 µm_ is calculated by integrating the spectral reflectance over the wavelength range of 8–13 µm and normalizing by the number of wavelength data points. APPHs exhibit the highest reflectance in the 8–13 µm range, while the APRC shows relatively lower reflectance, indicating greater absorptance. Accordingly, Figure S16 (Supporting Information) presents the correlation between structural parameters and optical characteristics. Unlike APTMs with 20 nm Ag layers, the 10 nm Ag layers annealed at 400 and 600 °C exhibit high solar absorption (*P_sun_*) while maintaining high reflectance in the LWIR region (*R_8‐13 µm_*), enabling operation as an effective APPH. On the other hand, the APTM with 20 nm Ag layers annealed at 1000 °C shows the lowest *P_sun_* and *R_8‐13 µm_*, making it suitable as an APRC. Additionally, our findings indicate that the colorimetric and thermal engineering functions (i.e., photothermal heating and radiative cooling) can operate independently as detailed in Note S5 (Supporting Information).

### 2.6 Anti‐Fogging Experiments and Durability Tests

Using photothermal properties, APPHs could be applied as defrosters for laminated glass in structural designs (Figure 5d). This illustration is included to highlight the potential applications of APPH and APRC in automotive glass for anti‐fogging. The combination of solar energy absorption and LWIR reflection enables APPHs to function as effective defoggers. Unlike the rear window, the front windshield typically lacks heating wires to avoid obstructing the driver's view.^[^ ^57^, ^58^ ^]^ The mechanism of commercial anti‐fogging coatings is based on modifying the surface wettability. Hydrophobic coatings have contact angles above 90° and form spherical water droplets that roll off easily but scatter light. In contrast, hydrophilic coatings show contact angles below 90° and spread water into a thin film that reduces light scattering. In both cases, mechanical supports such as wipers or inclined surfaces are typically required to physically remove the accumulated water film or droplets. However, photo‐induced heating functionality can be achieved by the APPH without any electrical energy, which ensures active temperature control, efficient defrosting, and de‐icing.^[^ ^59^, ^60^ ^]^ Due to the high solar absorptance and strong LWIR reflectance, APPHs efficiently convert sunlight into heat and retain a higher surface temperature compared to the surrounding environment. The outstanding photothermal heating capability promotes the rapid evaporation of condensed water, enabling continuous and self‐sustained fog removal that operates entirely without mechanical assistance. To evaluate the anti‐fogging performance of the APPH, an experimental setup is constructed in which hot water in a Styrofoam box generates moisture on the surface of the samples (Figure S17, Supporting Information). Figure 5e compares the defrosting process between bare soda lime glass and the APPH. After 25 seconds, the moisture completely disappears from the surface of the APPH, whereas it remains on the glass surface. Additionally, the video demonstrates the real‐time removal of moisture from the APPH (Video S1, Supporting Information). This highlights a robust defogging capability that outperforms previous photothermal anti‐fogging studies.^[^ ^61^, ^62^ ^]^ The large temperature difference of the APPH from the ambient temperature (above 18 °C) robustly prevents fogging under solar irradiation. We summarize the state‐of‐the‐art in evaporative defogging technologies in Table S3 (Supporting Information).^[^ ^62^, ^63^, ^64^, ^65^ ^]^ Among various reported studies, APPH demonstrates both high heating efficiency and cost‐effectiveness. The defrosting time of the APPH can be further reduced by incorporating a hygroscopic layer.

To evaluate the durability of the APTMs, environmental tests are conducted under UV exposure and high humidity conditions (Figures S18 and S19, Supporting Information). Even after 12 h under high humidity and 24 h of UV exposure, the optical properties of the APTMs are well preserved. To solve the mechanical durability problem of the APTMs, we introduce a coating material called “HU‐DP 200 (HANDOK, Korea)”, which is known for excellent mechanical robustness and environmental resistance. This coating layer has a minimal impact on the optical response across the solar and LWIR spectral regions (Figure S20, Supporting Information). After the coating process, the mechanical strength of the samples is evaluated through nanoindentation, which provides quantitative insights into hardness and elastic modulus. Table S4 (Supporting Information) summarizes the stiffness, hardness, reduced elastic modulus, and maximum indentation depth of the APPH and APRC samples with the coating. Nanoindentation tests revealed that both samples possess mechanical strength comparable to PMMA and PS, sufficient to withstand surface pressure and handling stress. In addition to mechanical robustness, the APTMs also exhibit strong fire resistance due to high‐temperature annealing during fabrication. Such thermal stability enhances durability, extends operational lifetime, and lowers fire risk in practical applications.

## 3 Conclusions

In summary, we propose a reconfigurable and effective heat control system utilizing the aesthetic properties of APTMs. APTMs are highly effective for thermal regulation due to ease of fabrication, cost‐effectiveness, large‐area scalability, and controllable optical properties. The optical characteristics of APTMs actively vary in both the solar spectrum and LWIR regions, depending on the configuration of the Ag layers. The dense Ag particles formed at low annealing temperatures contribute to high reflectance in the LWIR region, whereas higher annealing temperatures (e.g., 1000 °C) result in sparse Ag components and increased LWIR absorptance. Furthermore, the diverse colorimetric features of APTMs can be tuned by adjusting the thickness of each layer and the annealing temperature during the fabrication process. The wide color range of APTMs, attributed to plasmonic resonances within the structure, suggests potential for expanded applications. Our computational studies and outdoor measurements demonstrate optimized conditions for operating as a photothermal heater or radiative cooler. Additionally, the photothermal heater can act as an effective defroster based on the photo‐induced heating ability, which enables rapid moisture elimination and prevents condensation on the treated surface. As a result, APTMs overcome the limitations of conventional thermal regulators by expanding applications in vehicles and architectures, aided by aesthetic aspects and optical controllability. We believe that our innovative thermal engineering design can establish a new standard for efficient solar‐responsive thermal management systems.

## 4 Experimental Section

### Optical Simulation

Rigorous coupled‐wave analysis (RCWA) simulations were performed using DiffractMOD (RSoft Design Group, Synopsys, United States) to analyze the 2D electric field and absorption profiles of the APTM. To observe each distribution, a 2D monitor was placed at the center of the *xz*‐plane in the simulation domain. The domain size was set to 2 µm along both the *x*‐ and *y*‐axes, and a plane wave was used as the incident light. The effective medium theory (EMT) layer was used to model the bottom film to estimate the volume ratio between Ag and air. Data visualization was performed using OriginPro 2022 (OriginLab Co., United States) and the Python plotting library Matplotlib.

### Measurements

TEM (TALOS F200X, FEI, Netherlands), EDS (GEMINI500, Carl Zeiss, Germany), and SEM (SUPRA40, Carl Zeiss, Germany) were used to discover the nano‐structural features (i.e., diameter and fill factor) and compositional characteristics of the APTM. Reflection spectra in the visible region were obtained using a UV–vis‐NIR spectrophotometer (V‐770, JASCO Co., Japan). Optical efficiencies in the LWIR region were measured by FT‐IR (Vertex 70v, Bruker).

### Statistical Analysis

During the preprocessing of spectral data, values below 0% and above 100% were clipped to 0% and 100%, respectively. Analyses of discrete nanoparticle size and fill factor were conducted using the open‐source image processing software ImageJ. Using the particle analysis function in ImageJ, the total particle count, average diameter, size distribution, and fill factor of Ag particles was quantified.

### Extraction of Color Coordinates

The CIE 1931 color space has been developed to model human color perception system. In this color space, colors were represented based on tristimulus values *x*, *y*, and *z*, which were calculated using the following Eqs. (1), (2), and (3).

(1) $$x=100\frac{\int I\left(\lambda \right)R\left(\lambda \right)\bar{x}\left(\lambda \right)d\lambda }{\int I\left(\lambda \right)\bar{y}\left(\lambda \right)d\lambda }$$

(2) $$y=100\frac{\int I\left(\lambda \right)R\left(\lambda \right)\bar{y}\left(\lambda \right)d\lambda }{\int I\left(\lambda \right)\bar{y}\left(\lambda \right)d\lambda }$$

(3) $$z=100\frac{\int I\left(\lambda \right)R\left(\lambda \right)\bar{z}\left(\lambda \right)d\lambda }{\int I\left(\lambda \right)\bar{y}\left(\lambda \right)d\lambda }$$

where $\bar{x}\left(\lambda \right)$, $\bar{y}\left(\lambda \right)$, and $\bar{z}\left(\lambda \right)$ were the CIE color‐matching functions, *I*(λ) was the CIE D65 standard illuminant spectrum, and *R*(λ) was the spectral reflectance of the sample. Using tristimulus values, coordinates (*X*, *Y*) in the CIE 1931 color space of chromaticity could be derived as shown in Equation (4) and Equation (5):

(4) $$X=\frac{x}{x+y+z}$$

(5) $$Y=\frac{y}{x+y+z}$$

## Conflict of Interest

The authors declare no conflict of interest.

## Supporting information



Supporting Information

Supplemental Video 1

## Data Availability

The data that support the findings of this study are available from the corresponding author upon reasonable request.
