# Supplementary material for: Color‐Tunable Glass for Adaptable Thermal Management Based on Silver Phase Change
Source: Adv Sci (Weinh). 2025 Jun 10;12(34):e05791. doi: 10.1002/advs.202505791 (PMC12442692; doi:10.1002/advs.202505791)
Supplement: Supplementary file 1 — Supporting Information [file ADVS-12-e05791-s002.pdf]

ADVANCED SCIENCE
Open Access

Supporting Information

for *Adv. Sci.*, DOI 10.1002/advs.202505791

Color-Tunable Glass for Adaptable Thermal Management Based on Silver Phase Change

Mi Jin Hong, Seon Kyeong Kim, Jung Mi Im, Ik Hoon Jeong, Min Ju Kim, Jeong Jin Kim, Yeong Jae Kim and Gil Ju Lee**

Supporting Information

Color-Tunable Glass for Adaptable Thermal Management Based on Silver Phase Change

Mi Jin Hong[†,1], Seon Kyeong Kim[†,2], Jung Mi Im[1], Ik Hoon Jeong[1], Min Ju Kim[1], Jeong Jin Kim[1], Yeong Jae Kim*[,2], and Gil Ju Lee*[,1]

[1]School of Electrical and Electronics Engineering, Pusan National University, 2, Busandaehak-ro 63 beon-gil, Geumjeong-gu, Busan, Republic of Korea, 46241.

[2]Ceramic Total Solution Center, Korea Institute of Ceramic Engineering and Technology, 3321, Gyeongchung-daero, Sindun-myeon, Icheon-si, Gyeonggi-do 17303, Republic of Korea

*Correspondence to: gjlee0414@pusan.ac.kr, kimyj@kicet.re.kr

† These authors equally contributed to this work

Supporting Notes

Supporting Note 1. Maxwell-Garnett effective medium theory (EMT) for optical simulation

A particle-embedded film containing various particles can be treated as a homogeneous medium when the particle size is much smaller than the wavelength of light. The effective complex refractive index of the medium can be determined using the Maxwell-Garnett effective medium theory, as described in Eq. (1)[1]. In this equation, epsilon (ε) represents the effective dielectric constant to be calculated. ε_0 denotes the dielectric constant of the host material, ε_i refers to the dielectric constant of the inclusions, and η_i corresponds to the volume fraction of each inclusion.

$$\frac{\varepsilon-\varepsilon_0}{\varepsilon+2\varepsilon_0} = \sum_i \eta_i \frac{\varepsilon_i-\varepsilon_0}{\varepsilon_i+2\varepsilon_0} \qquad (1)$$

The effective complex refractive index is obtained by establishing the relationship between the refractive index and the dielectric constant, as shown in Eqs. (2) and (3).

$$n = \sqrt{\frac{1}{2}\left(\sqrt{\varepsilon_{real}^2 + \varepsilon_{imaginary}^2} + \varepsilon_{real}\right)} \qquad (2)$$

$$k = \sqrt{\frac{1}{2}\left(\sqrt{\varepsilon_{real}^2 + \varepsilon_{imaginary}^2} - \varepsilon_{real}\right)} \qquad (3)$$

Since we use the Maxwell-Garnett EMT in the LWIR, where the effects of nanoparticles are negligible, the simulation results fit well in the LWIR region. In this demonstration, Ag is treated as a homogeneous host material, while air serves as the inclusions.

Supporting Note 2. Angle tolerance of the colorimetric performance achieved through the TiO_2 intermediate layer

The presence of the intermediate TiO_2 layer also enhances the isotropic colorimetric properties. A schematic illustration of the phase shifts corresponding to each term in Eq. (1) is provided in **Figure SN1**. The schematic depicts the incident light being reflected at both the upper and lower metal interfaces. The total reflection coefficient is defined as the amplitude ratio of E_r to E_i. Phase shifts occur both at the reflection points and during propagation through the dielectric layer. The resonance condition can be expressed as shown in Eq. (1):

$$\emptyset r_{21} + \emptyset r_{23} + \emptyset r_{prop} = 2m\pi \qquad (1)$$

where ϕ_{r21} and ϕ_{r23} represent the phase shifts induced by internal reflections at the metal-insulator interfaces, and ϕ_{prop} indicates the propagation phase shift associated with the optical path difference through the cavity. Here, m is a positive integer corresponding to the order of resonance. In a Fabry-Perot cavity, maintaining the resonance condition under varying angles of incidence is crucial for preserving stable optical performance, such as color appearance. However, an increase in the incident angle changes the optical path length within the cavity and consequently modifies ϕ_{prop}, which can disturb the resonance condition.

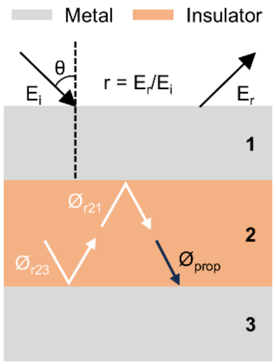

Figure SN1. Schematic of Ag-TiO_2-Ag (MIM) resonator and phase shift of incident light.

High refractive index dielectrics such as TiO_2 can minimize this effect. Since the increase in optical path length is smaller in a high-index medium, the phase shift changes less with angle than in low-index materials like SiO_2[2]. As a result, TiO_2-based cavities exhibit significantly better angular tolerance, minimizing resonance wavelength shifts even at oblique incidences (**Figure SN2**). This difference is critical for practical applications, ensuring that the visible color generated by the cavity remains stable across a wide range of viewing angles, which is essential for maintaining consistent aesthetic and optical performance.

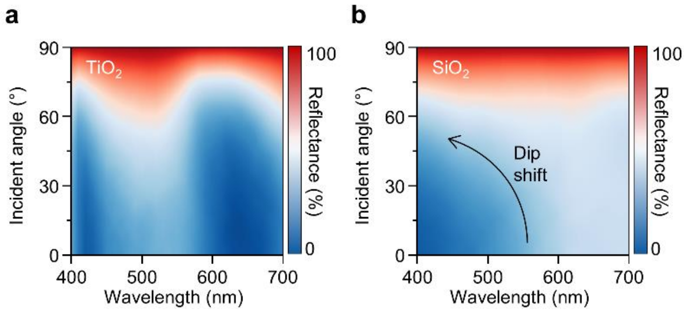

Figure SN2. Angle-dependent reflectance in the visible region for samples with TiO_2 and SiO_2 as intermediate layers.

Supporting Note 3. The influence of asymmetric Ag thickness on optical characteristics

To investigate the impact of asymmetry in Ag layers, we additionally fabricated samples using combinations of 5 nm, 10 nm, and 20 nm thicknesses, as employed in our study. The TiO_2 thickness is fixed at 150 nm. The resulting six configurations and their corresponding visible colors are summarized in **Table SN1**. When the thicker Ag layer is positioned on the bottom side, the color varies from blue to greenish. In contrast, when the thicker layer is on the top side, the color remains relatively consistent. **Figure SN1** presents the optical response in the solar spectrum, and **Table SN2** summarizes the power density of solar irradiance across the relevant wavelength range. FT-IR results further confirm that a thicker Ag layer on the bottom side significantly enhances reflectance in the LWIR region (**Figure SN2**). Among the various combinations, the configuration with 5 nm Ag on the top side and 10 nm Ag on the bottom side is identified as the most suitable for APPH operation, as it provides both the highest absorbed solar energy and strong LWIR reflectance. These findings indicate that the thickness of both Ag layers plays a critical role in optimizing colorimetric and infrared functionalities.

	Raw	400 °C	600 °C
Ag 10 nm (Top) / Ag 5 nm (Bottom)	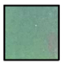	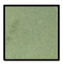	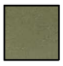
Ag 20 nm (Top) / Ag 5 nm (Bottom)	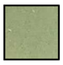	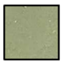	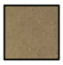
Ag 20 nm (Top) / Ag 10 nm (Bottom)	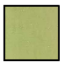	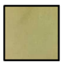	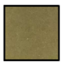
Ag 5 nm (Top) / Ag 10 nm (Bottom)	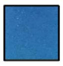	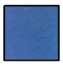	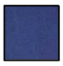
Ag 5 nm (Top) / Ag 20 nm (Bottom)	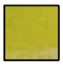	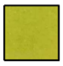	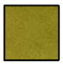
Ag 10 nm (Top) / Ag 20 nm (Bottom)	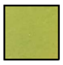	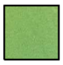	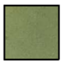

Table SN1. Photographs of samples with asymmetric Ag layers on both sides.

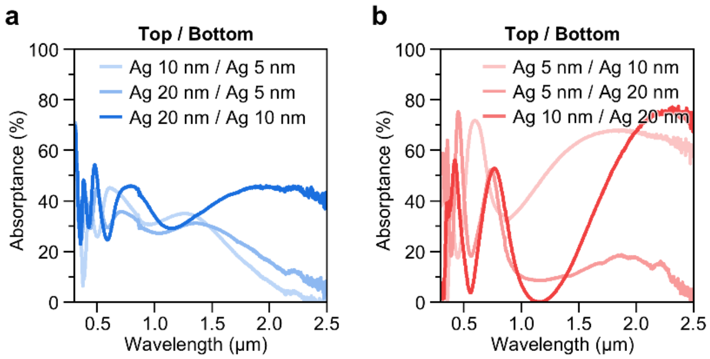

Figure SN1. Absorptance in the solar spectrum for (a) samples with the thicker Ag layer on the top side, and (b) samples with the thicker Ag layer on the bottom side.

	Ag 10 nm / Ag 5 nm	Ag 20 nm / Ag 5 nm	Ag 20 nm / Ag 10 nm	Ag 5 nm / Ag 10 nm	Ag 5 nm / Ag 20 nm	Ag 10 nm / Ag 20 nm
P_{sun}	33.2 W/m^2	31.8 W/m^2	37.9 W/m^2	40.4 W/m^2	19.1 W/m^2	22.7 W/m^2

Table SN2. Absorbed solar energy for each Ag thickness combination.

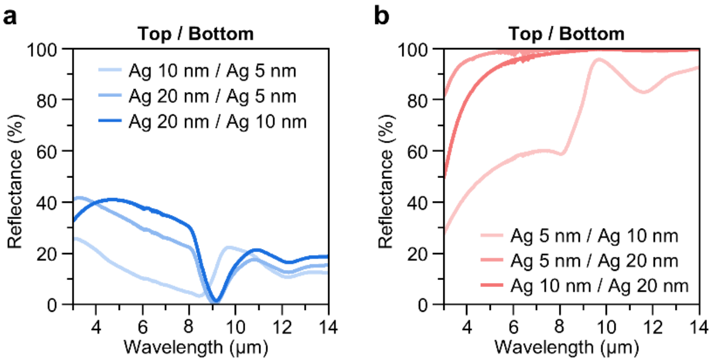

Figure SN2. FT-IR spectra of the six configurations: (a) thicker Ag layer on the top side, (b) thicker Ag layer on the bottom side.

Supporting Note 4. Description of heat transfer simulation

In this section, we present a detailed method for heat transfer simulation, as shown in **Figure 4c and Figure 4d**. The simulation conditions are listed in **Table SN1,** and the size specifications of the components are illustrated in **Figure SN1**. Beneath the APTM, polyurethane (PU) is placed inside an acrylic box to analyze temperature changes in the inherent component affected by the APTM. **Figure SN1b** exhibits a heat transfer model in which a solid-to-solid radiation model is applied to all components within the simulation domain. However, the surface-to-surface heat transfer is enabled only between the upper and lower sides of the APTM and the top surface of the PU.

Parameters	Values
Initial temperature of solids	293.15 K
External temperature	293.15 K
Solar irradiance	1000 W/m^2
Heat transfer coefficient (h_c)	10 W/(m^2·K)
Object properties	Materials from *COMSOL* material library
Acrylic box	Acrylic plastic
APTM devices	Clear soda lime silica
Polyurethane	Polyurethane (solid)

Table SN1. The parameters used in the heat transfer simulation. The parameters and object properties in the simulation are described. In particular, the heat transfer coefficient is set to 10 W/(m^2·K) to account for both heat conduction and convection. The sun's position is modeled based on the specific location of Seoul, South Korea, on January 1, 2025. Materials for each object are assigned from the *COMSOL* material library.

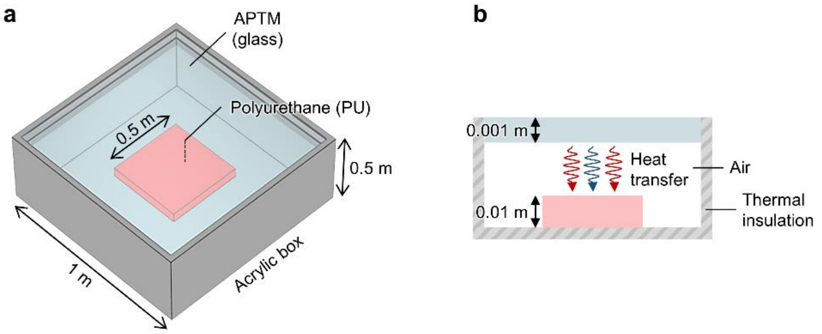

Figure SN1. The computational domain and components for physics simulation. (a) The schematic of the simulation domain consisting of an acrylic box, APTM, and polyurethane (PU). The size information of each structure is also depicted. (b) A cross-sectional view of the domain. The acrylic box is considered a thermal insulator, whereas heat flux is applied to the APTM and the PU. Therefore, surface-to-surface radiation and heat transfer between the APTM and the PU can be observed.

The ambient temperature is modeled as a cosine function with an average value of 20 °C and an amplitude of 3 °C over two days (**Figure SN2a**). The sky transmittance is set according to **Figure SN2b**. To evaluate the thermal management performance of APTMs, the emissivity in the 0.3-16 μm range under various APTM conditions is inserted into the soda lime glass from the *COMSOL* library, based on values derived from absorptance in the LWIR region.

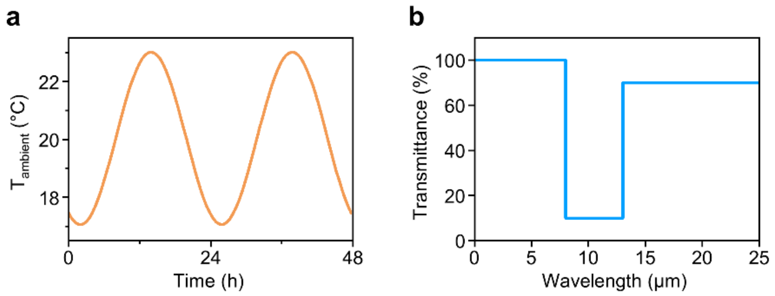

Figure SN2. The parameters used in physics simulation. (a) The ambient temperature is modeled as a sinusoidal function of time over two days, shown in degrees Celsius. The average temperature is set at 20 °C, with a half-diurnal temperature variation of 3 °C. (b) The sky transmittance within the spectral band of the LWIR region. The transmittance is 100% for the 0-8 μm range, while the 8-13 μm and 13-25 μm ranges have transmittances of 30% and 90%, respectively.

Supporting Note 5. Color appearance independent of infrared functionalities

The color of APTMs may be inherently limited by the functional selection (*i.e.,* photothermal heating and radiative cooling) in the LWIR region. However, our findings indicate that the colorimetric and thermal engineering functions can operate independently. In the cavity-resonant system, the thickness of the dielectric (TiO_2) layer determines the dominant visible color[3-5]. As displayed in **Figure SN1**, color variation occurs as the TiO_2 thickness increases from 50 nm to 150 nm. **Figure SN2** shows the corresponding CIE 1931 color coordinates. To examine this effect, we fabricated additional samples with TiO_2 thicknesses of 90, 130, and 150 nm and analyzed optical characteristics in both the solar spectrum and the LWIR region (**Figure SN3**). Each thickness represents yellow, magenta, and cyan, respectively.

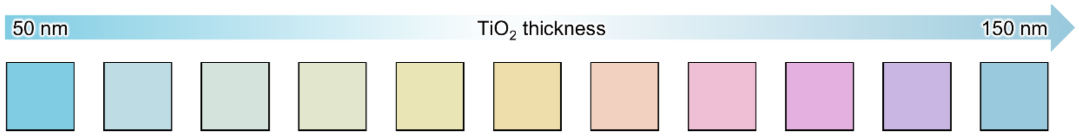

Figure SN1. Color variations as the TiO_2 thickness increases from 50 nm to 150 nm.

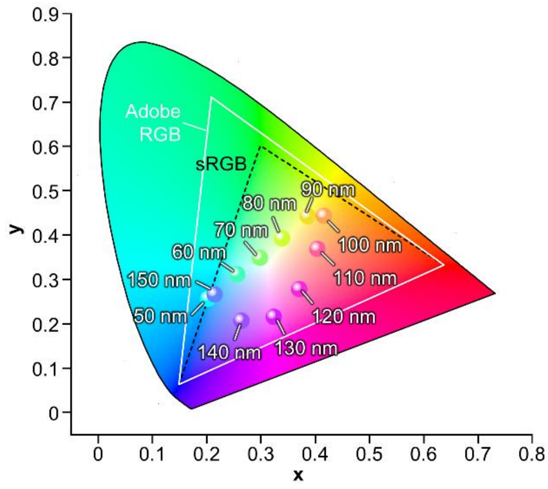

Figure SN2. CIE 1931 color coordinates as a function of increasing TiO_2 thickness.

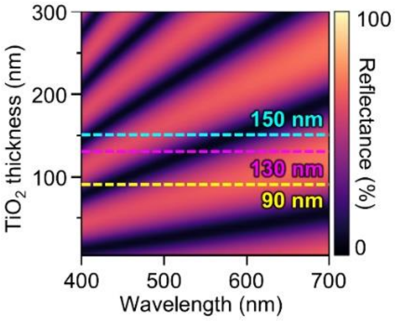

Figure SN3. Reflectance results in the visible region with increasing TiO_2 thickness. TiO_2 thicknesses of 90 nm, 130 nm, and 150 nm correspond to yellow, magenta, and cyan colors, respectively.

Using UV-Vis-NIR spectrophotometry and FT-IR spectroscopy, we measured the optical characteristics of the APTMs with varying TiO_2 thicknesses. These samples were also annealed at 600 °C to mimic the structural and optical properties of the APPH. As shown in **Figure SN4**, the absorptance in the solar region varies with the thickness of the TiO_2. The photographs above display the actual colors of the APPHs, which closely match the simulation results, indicating distinct hues of yellow, magenta, and cyan. The sample with 150 nm TiO_2 corresponded to the APPH and exhibits a P_{sun} value of 35.5 W/m². Despite variations in sample color, the absorbed solar power remains well preserved as the TiO_2 thickness changes, demonstrating stable operation of the APPH (**Table SN1**). Furthermore, FT-IR results confirm that the high reflectance in the LWIR region is maintained and does not degrade the photothermal performance (**Figure SN5**). Therefore, the TiO_2 thickness effectively tunes the colorimetric properties without diminishing IR functionalities.

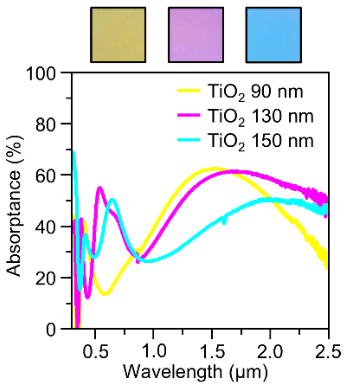

Figure SN4. UV-Vis-NIR spectrophotometer results of APTMs with TiO_2 thicknesses of 90 nm, 130 nm, and 150 nm.

TiO_2 thickness	90 nm (Yellow)	130 nm (Magenta)	150 nm (Cyan)
P_{sun}	29.9 W/m^2	39.1 W/m^2	35.5 W/m^2

Table SN1. Absorbed solar power for TiO_2 thicknesses of 90, 130, and 150 nm.

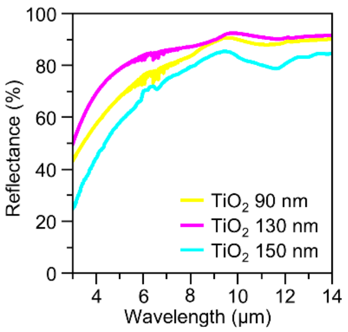

Figure SN5. FT-IR results of APTMs with TiO_2 thicknesses of 90 nm, 130 nm, and 150 nm.

Also, changes in the TiO_2 thickness could potentially affect the radiative cooling performance. **Figure SN6** shows the absorptance in the solar spectrum and the reflectance spectra in the 3-14 μm range for APTMs with TiO_2 thicknesses of 90 nm, 130 nm, and 150 nm annealed at 1000 °C. The combination of low absorbed solar energy and low reflectance in the LWIR region enables effective radiative cooling. These findings are consistent with computational results shown in **Figure SN7**, where changes in TiO_2 thickness do not alter absorptance or reflectance in the LWIR region. thereby reinforcing the functional independence of color and thermal performance. Experimental observations confirm that the visible colors of APTMs are independent of their thermal function, whether the device functions as a photothermal heater or a radiative cooler, proving that color and IR functionality operate independently.

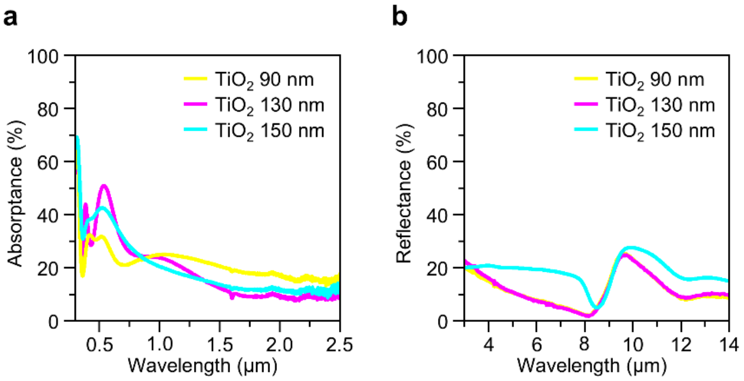

Figure SN6. FT-IR results of APTMs with TiO_2 thicknesses of 90 nm, 130 nm, and 150 nm.

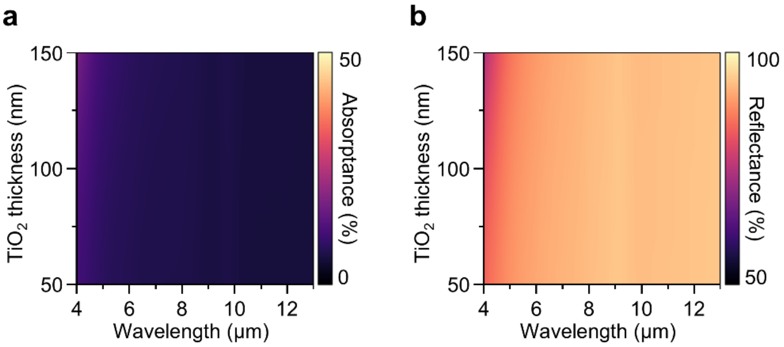

Figure SN7. Optical characteristics of APTMs with increasing TiO_2 insulating layer thickness in the LWIR region.

The angle-dependent coloration effects of APTMs are critical to apply practical and aesthetic considerations in real-world applications. Therefore, we included photographs and conducted additional computational analyses to demonstrate the isotropic coloration performance of APTMs. As shown in **Figure SN8**, we captured images of the APTMs with colors corresponding to sky-blue, magenta, and yellow at viewing angles ranging from 0° to 70°. The color of the APTMs can be controlled by varying the thickness of the insulating TiO_2 layer, with cyan, magenta, and yellow achieved at thicknesses of 150 nm, 130 nm, and 90 nm, respectively. The original color of each sample is preserved up to a viewing angle of 50-60°. Instead of varying the incident angle, we fabricated a setup using a 3D printer to evaluate the angle robustness of the samples (**Figure SN9**). The camera is positioned in the normal direction above the setup to capture the reflected colors.

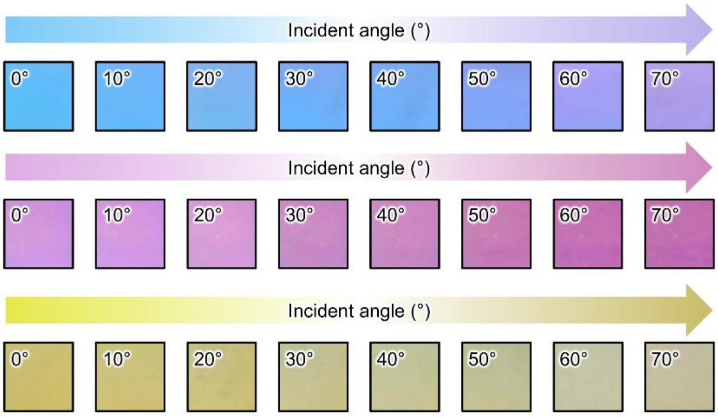

Figure SN8. Color variation depending on the viewing angle of the samples, displaying sky-blue, magenta, and yellow hues. Photographs were captured to demonstrate the angle robustness of the APTMs.

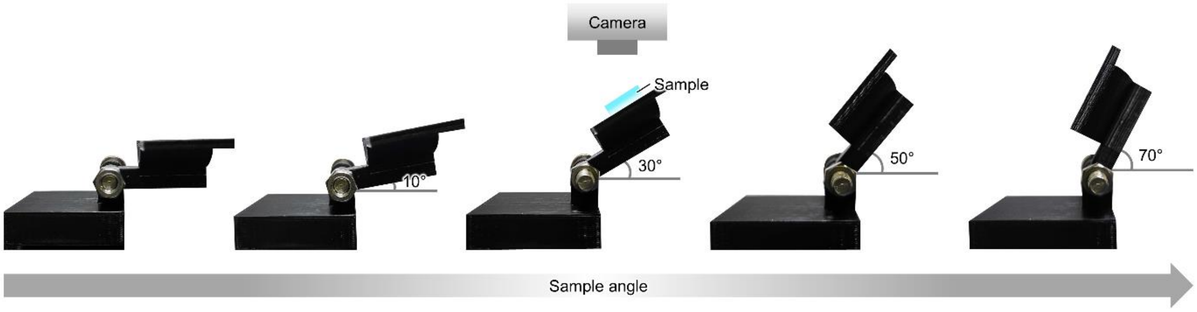

Figure SN9. A setup for capturing photographs of samples at various viewing angles, with a cross-sectional view showing angles of 0°, 10°, 30°, 50°, and 70°.

Supporting Tables

$* \ P_{sun} = \int_0^\infty I_{AM1.5G}(\lambda)\epsilon_{sample}(\lambda)d\lambda$

P_{sun} (W/m^2)	Ag 10 nm	Ag 20 nm
400 °C	APPH 40.7	15.1
600 °C	APPH 35.5	31.0
1000 °C	23.5	APRC 14.2

Table S1. The absorbed power density of solar irradiance in the solar spectrum. The power density of solar irradiance (P_{sun}) is calculated as the integral of the absorbed solar intensity, which is obtained by multiplying the AM1.5G solar spectrum by the sample's absorptance as a function of wavelength. The equation for the absorbed power density is presented in the table. The solar spectrum ranges from 0.25 µm to 2.5 µm.

$R_{8\text{-}13\mu m}$ (%)	Ag 10 nm	Ag 20 nm
400 °C	APPH 83.9	99.9
600 °C	APPH 82.1	99.1
1000 °C	11.9	APRC 17.8

Table S2. Average reflectance of the APTMs in the 8-13 μm region. The average reflectance in the LWIR region was calculated under different fabrication conditions. The APPHs exhibit the highest reflectance, whereas the APRC shows a significantly lower value.

Refs	Structure	Heating efficiency (under 1 sun)	Defogging time	Cost-effectiveness
[62]	Au metamaterial sandwiched by TiO_2 layers	+8.3 °C	25 seconds	$1.97/cm^2
[63]	Repeated ultrathin bilayers of Au nanoparticles and a TiO_2 nanolayer	+3 °C	70 seconds	$1.39/cm^2
[64]	Cesium-doped tungsten trioxide (CWO) and benzotriazole (BTA) nanoparticles embedded in resin	+38 °C	60-80 seconds	$1.06/cm^2
[65]	Cs_xWO_3 nanorods and PVA-Zn hygroscopic layer	+24.6 °C	14 seconds	$1.82/cm^2
Ours	Ag-TiO_2-Ag layers on a soda-lime glass	+18.1 °C	25 seconds	$0.54/cm^2

Table S3. A comparison of photothermal performance and cost-effectiveness between our device and previous studies. The anti-fogging performances, including heating efficiency, defogging time, and cost-effectiveness, are compared with those of photothermal method-based anti-fogging strategies.

	APPH	APRC
Stiffness (mN/μm)	120.20	97.931
Hardness (MPa)	161.91	119.91
Reduced Elastic Modulus (MPa)	2802.0	2528.3
Max Depth (μm)	0.13649	0.15751

Table S4. Nanoindentation measurement results of the APPH and APRC coated with HU-DP 200. The APPH exhibits an average hardness of 161.9 MPa and a reduced elastic modulus of 2.80 GPa, whereas the APRC shows slightly lower values of 119.9 MPa and 2.53 GPa, respectively. In addition, the APPH shows higher stiffness (120.2 mN/μm) and a shallower maximum indentation depth (0.136 μm) than the APRC (97.9 mN/μm, 0.158 μm), indicating greater elastic resistance and reduced susceptibility to deformation.

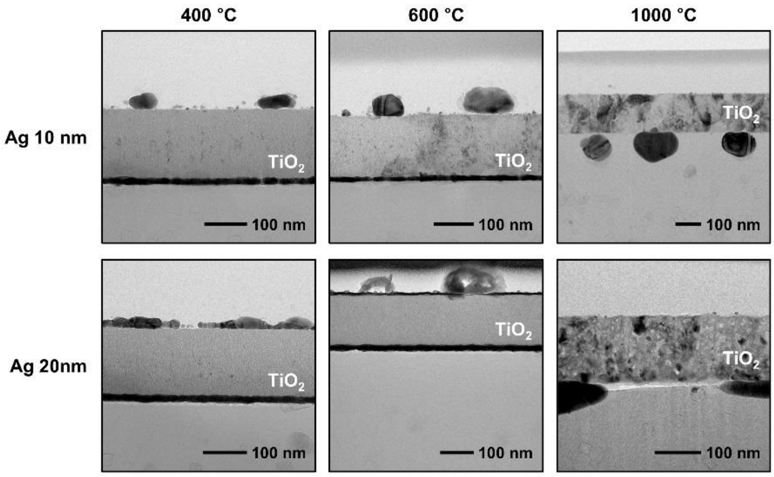

Figure S1. TEM analysis of APTMs under various conditions. TEM results show the cross-sectional morphologies of APTMs. As the annealing temperature increases, the top Ag layer gradually transforms into nanoparticles at lower temperatures, whereas the bottom Ag layer undergoes dewetting at higher temperatures.

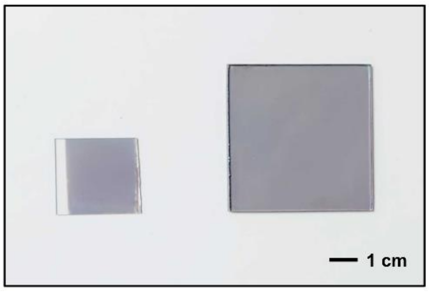

Figure S2. Large-scale fabrication of the APTM. The APTM can be fabricated on a large scale over a 2-inch substrate (right) due to the simplicity and reproducibility of the fabrication process.

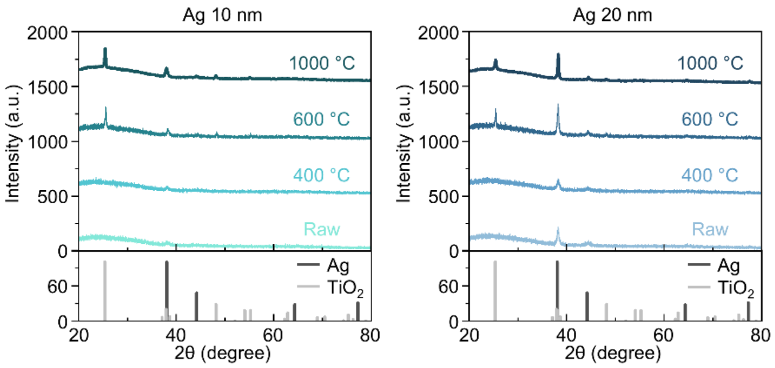

Figure S3. X-ray diffraction (XRD) results of the APTMs. XRD results of the APTMs with Ag layers of 10 nm and 20 nm, listed according to the annealing temperature. Ag peaks emerge when the Ag layer thickness is 20 nm. Above 400 °C, the amorphous TiO_2 phase transforms into an anatase TiO_2. From the XRD results, we can conclude that high temperatures during the annealing process do not affect the composition of each layer.

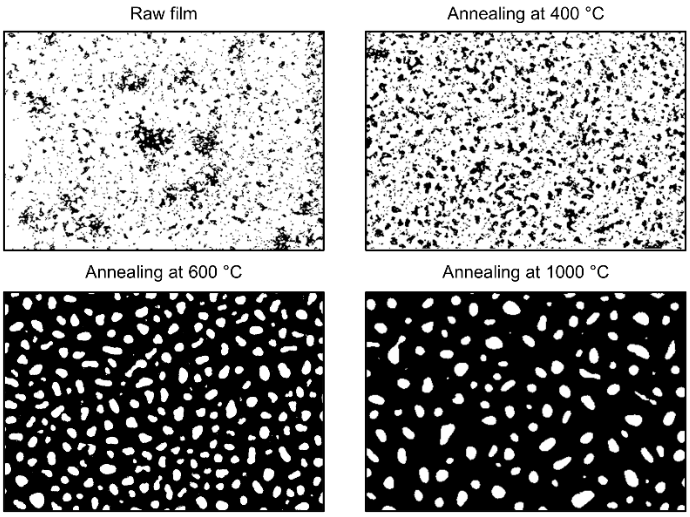

Figure S4. The threshold results in SEM images according to the annealing temperature. The threshold in SEM images was used to analyze the structural parameters of the top Ag layer. The threshold process enhances the contrast between areas covered by Ag NPs and the rest of the scanned domain in SEM images. As the annealing temperature increases, the fill factor of Ag NPs decreases sharply.

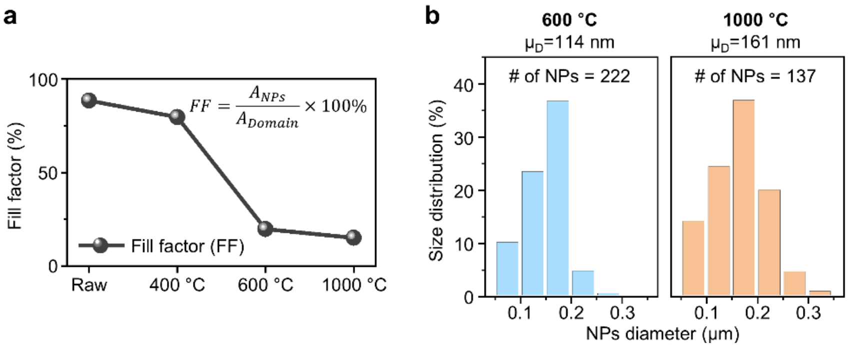

Figure S5. Structural analysis results of Ag NPs according to the annealing temperature for APTMs with a 10 nm Ag layer. (a) The fill factors of each sample, calculated from SEM images. Above an annealing temperature of 400 °C, the fill factor sharply decreases, while the raw sample and the sample annealed at 400 °C maintain a high fill factor above 75%. (b) The size distribution of Ag NPs in the APTMs annealed at 600 °C and 1000 °C. The former has a smaller mean diameter, with a more uniform size distribution.

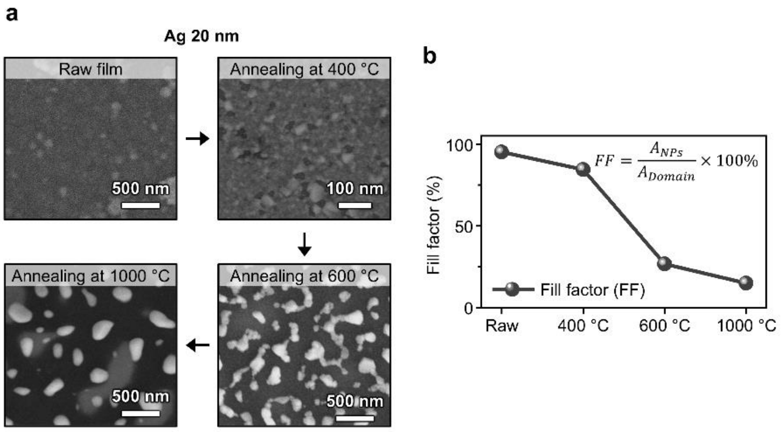

Figure S6. Structural analyses results of Ag NPs according to the annealing temperature for APTMs with a 20 nm Ag layer. (a) Top-view SEM images of the APTMs at different annealing temperatures. (b) The fill factor, calculated as the area ratio of NPs-covered to the total scanned region. (c) The size distribution of Ag nanoparticles annealed at 600 °C and 1000 °C.

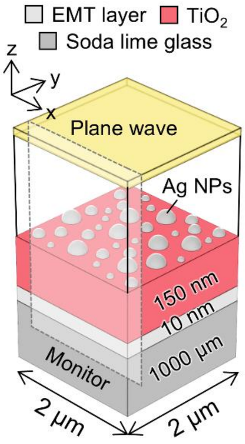

Figure S7. The simulation domain of the APTM. The three-dimensional model represents the APTM device, which consists of Ag NPs, a TiO_2 film, and a bottom layer modeled using EMT. The substrate is a 1000 μm-thick soda-lime glass, and the simulation domain area is set to 2 × 2 μm^2.

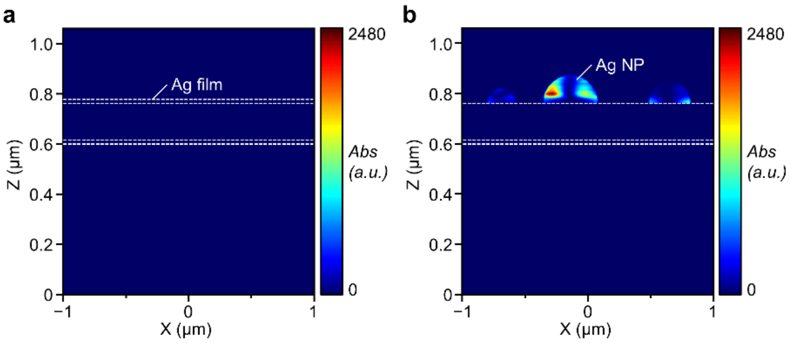

Figure S8. Absorption fields of the bare and nanostructured configurations. Two-dimensional absorption profiles at a wavelength of 2.0 μm for (a) Ag-TiO_2-Ag films and (b) Ag nanoparticles formed on the top surface.

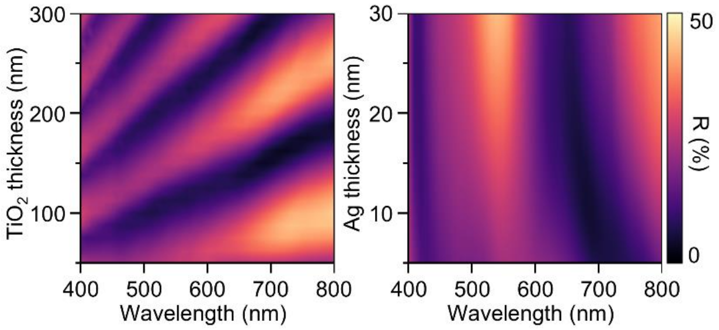

Figure S9. Simulation results for sweeping TiO_2 thickness and Ag layer thickness. The visible light reflectance depends on the thicknesses of the TiO_2 and Ag layers. The TiO_2 layer determines the peak position, whereas the Ag layer governs the bandwidth of the reflectance peak.

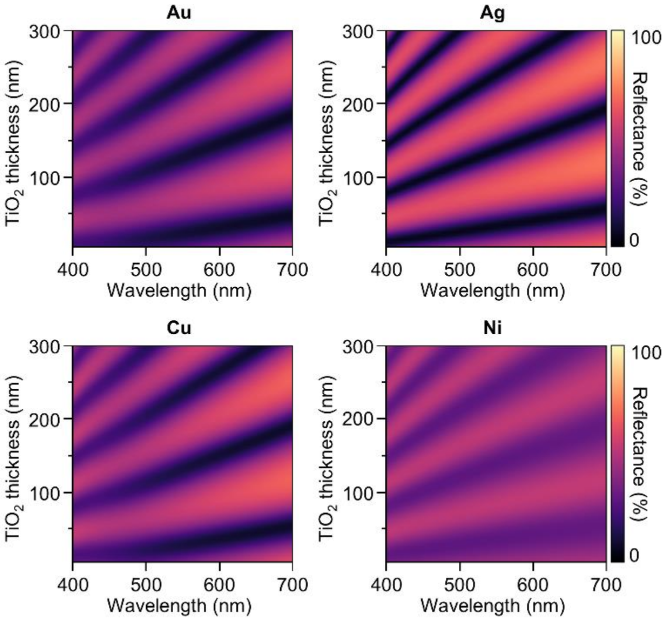

Figure S10. Reflectance results based on the TiO_2 thickness for various metal nanoparticles on the top side. Compared to Au, Cu, and Ni, Ag exhibits the most distinctive optical properties, as indicated by the distinguishable dips in the reflectance spectra.

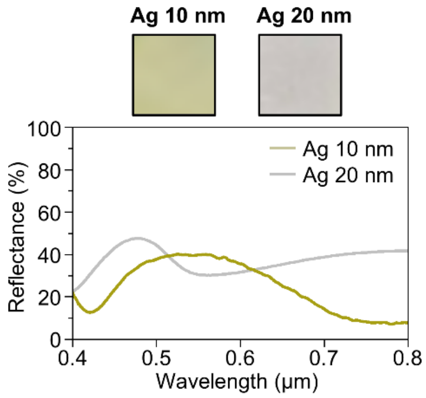

Figure S11. Optical characteristics in the visible region of APTMs annealed at 1000 °C. UV-Vis-NIR spectrophotometer results for APTMs annealed at 1000 °C with 10 nm and 20 nm Ag layers. The photographs above the graph show the visible colors of the APTM samples.

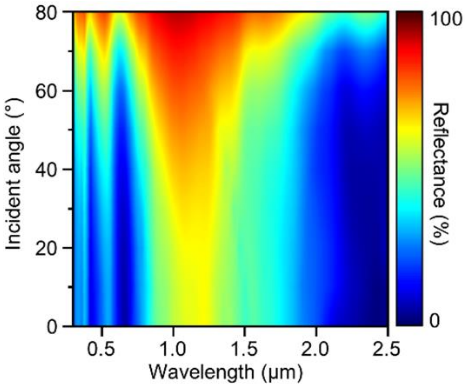

Figure S12. Angle robustness of APTMs. Reflectance spectra of the APTMs simulated at incident angles ranging from 0° to 80° within the solar spectrum.

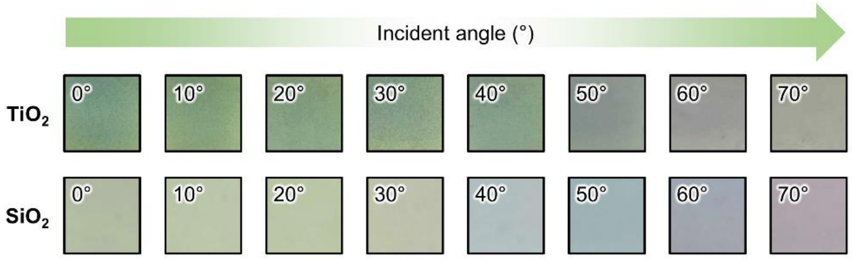

Figure S13. Angle-tolerant properties of TiO_2-based APTMs. Comparison of angular robustness between samples with TiO_2 and SiO_2 intermediate layers.

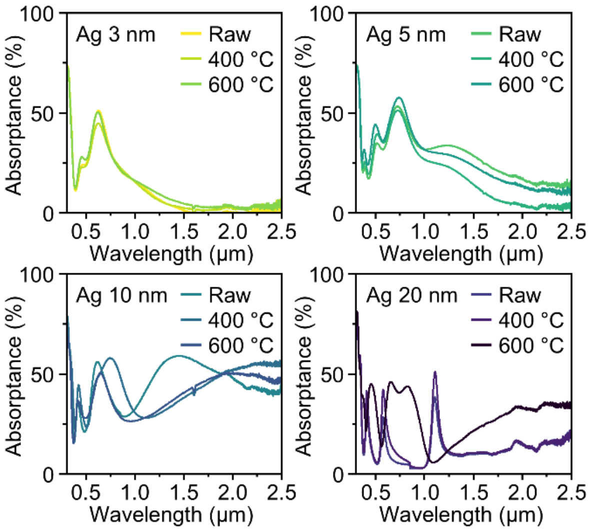

Figure S14. Measured absorptance results in the solar spectrum. The absorptance of the APTMs with 3, 5, 10, and 20 nm Ag layers was measured within the solar spectrum range (*i.e.,* 0.3-2.5 μm), derived from reflectance and transmittance data. The absorptance varies significantly with annealing temperature, particularly for the 10 nm and 20 nm Ag layers. High absorptance within the solar spectrum indicates increased heat generation due to the intense energy of solar radiation.

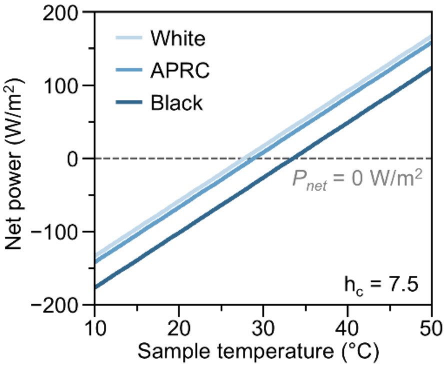

Figure S15. Net cooling power highlights the trade-off between aesthetics and infrared functionalities. The white and black samples represent ideal cases with visible absorptance values of zero and one, respectively, while all other optical parameters are identical to those of the APRC. The colored APRC, representing an intermediate state between white and black, shows cooling performance closer to that of the white sample, indicating that enhancing aesthetic appearance can reduce radiative cooling efficiency.

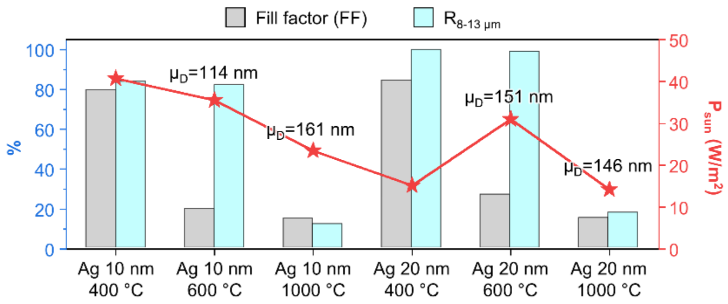

Figure S16. The relationship between nanoparticle morphologies and optical characteristics. To investigate the correlation between Ag morphology and optical performance, each parameter was analyzed. As the annealing temperature increases, both the fill factor and the reflectance in the LWIR region decrease. The sample with a 10 nm Ag layer annealed at 600 °C exhibits the highest absorbed solar energy, corresponding to the presence of the smallest and densest distribution of nanoparticles.

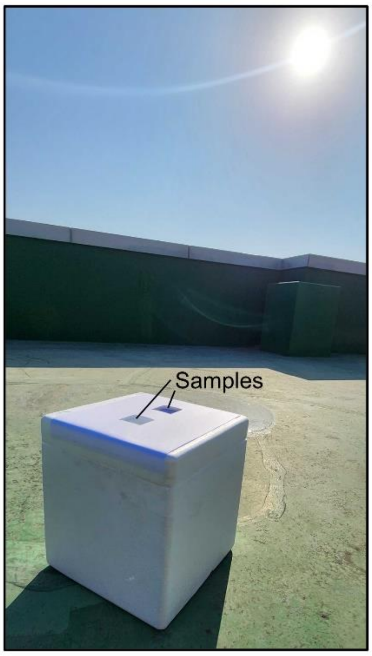

Figure S17. Anti-fogging experiment setup. To observe the de-fogging capability of the APPH, we set up the experiment in which the samples are placed under solar irradiation. Hot water in the Styrofoam box generates moisture on the surface of the samples.

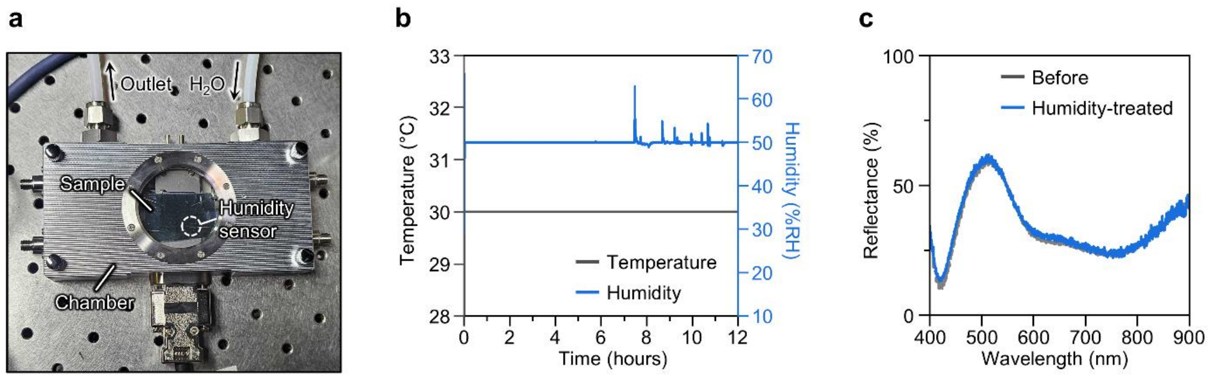

Figure S18. Durability tests under humid conditions. (a) Experimental setup for evaluating the humidity resistance of APTMs. (b) Temperature and humidity conditions are consistently maintained for 12 hours during the test. (c) Reflectance spectra before and after exposure to humidity.

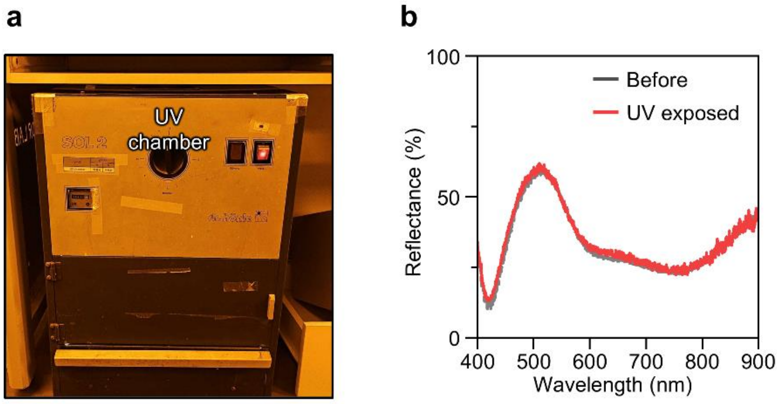

Figure S19. Durability tests under UV exposure. (a) UV chamber used for testing durability under UV irradiation. (b) Reflectance spectra of the APTM before and after 24-hour UV exposure.

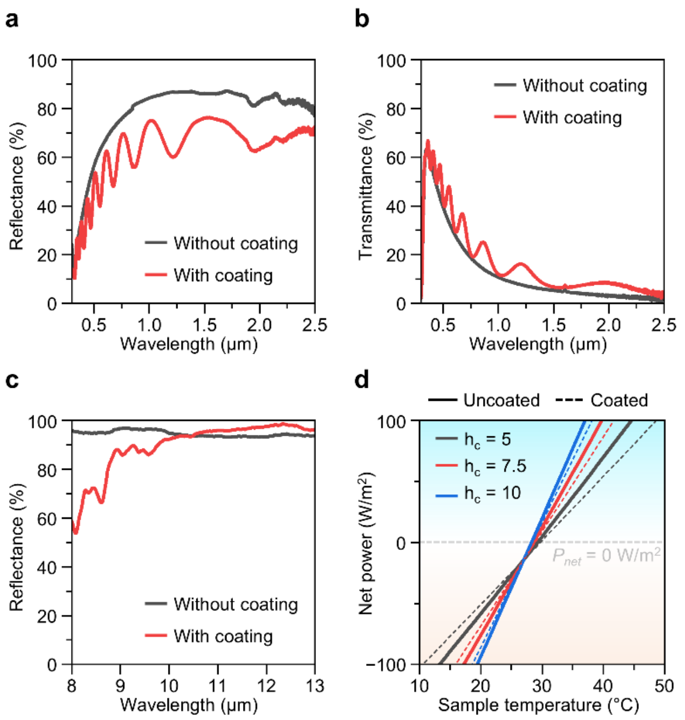

Figure S20. Optical responses of APTMs before and after HU-DP 200 coating. (a) Reflectance and (b) transmittance spectra of the samples with and without the HU-DP 200 coating layer in the solar spectrum. (c) Reflectance spectra in the LWIR region before and after passivation. (d) Net cooling efficiency of both cases, which is shown for heat transfer coefficients of 5, 7.5, and 10 W/m^2K.

References

[1] T. C. Choy, *Effective Medium Theory*, **2015**.

[2] D. H. Seo, S.-Y. Heo, D. H. Kim, Y. M. Song, G. J. Lee, *IEEE photonics journal (CD-ROM)* **2022**, *14*, 1.

[3] Z.-M. Yang, et al., *Advanced Optical Materials* **2016**, *4*, 1196.

[4] D. H. Kim, Y. J. Yoo, J. H. Ko, Y. J. Kim, Y. M. Song, *Optical Materials Express* **2019**, *9*, 3342.

[5] A. C. Kosger, A. Ghobadi, A. R. Rashed, H. Caglayan, E. Ozbay. *Optics letters* **2021,** *46*, 3464.
